# Prevalence and antimicrobial susceptibility of *Escherichia coli* O157 in beef at butcher shops and restaurants in central Ethiopia

**DOI:** 10.1186/s12866-017-0964-z

**Published:** 2017-03-03

**Authors:** Ashenafi Feyisa Beyi, Akafete Teklu Fite, Ephrem Tora, Asdesach Tafese, Tadele Genu, Tamirat Kaba, Tariku Jibat Beyene, Takele Beyene, Mesula Geloye Korsa, Fanos Tadesse, Lieven De Zutter, Bruno Maria Goddeeris, Eric Cox

**Affiliations:** 1 College of Veterinary Medicine and Agriculture, Addis Ababa University, Bishoftu, Ethiopia; 2 Department of Animal Sciences, University of Florida, Gainesville, FL USA; 3 Holetta Research Center, Ethiopian Institute of Agricultural Research, Holetta, Ethiopia; 4 Business Economics Group, Wageningen University, Hollandseweg 1, 6706 KN Wageningen, The Netherlands; 5 The Asia-Pacific Centre for Animal Health, Faculty of Veterinary and Agricultural Sciences, The University of Melbourne, Parkville, VIC Australia; 6 Faculty of Veterinary Medicine, University of Gent, Ghent, Belgium; 7 Division Animal and Human Health Engineering, Katholieke Universiteit Leuven, Leuven, Belgium

**Keywords:** Antimicrobial susceptibility, Beef, Butcher shops, *Escherichia coli* O157, Minced beef, Restaurants

## Abstract

**Background:**

Ethiopia bears the largest burden of foodborne diseases in Africa, and diarrheal diseases are the second leading causes of premature deaths. Enterohemorrhagic *Escherichia coli* O157 causes an asymptomatic infection to severe diarrhea and/or hemolytic-uremic syndrome in humans.

**Methods:**

A total of 440 beef carcass and in-contact surface swabs from 55 butcher shops and 85 minced beef samples from 40 restaurants in central Ethiopia were collected and examined for the presence of *E. coli* O157. Standard microbiological methods were used to isolate and identify *E. coli* O157 and to characterize the antimicrobial resistance of the isolates.

**Results:**

*E. coli* O157 was detected in 4.5% carcass swabs (*n* = 5) and 3.6% cutting board swabs (*n* = 4) samples from butcher shops. *E. coli* O157 was not detected in any of the minced beef samples obtained from restaurants. All isolates (*n* = 9) were 100% susceptible to five drugs, but five isolates were resistant to amoxicillin, two isolates to streptomycin and three isolates to chloramphenicol. One isolate was resistant to two drugs and another to three drugs.

**Conclusions:**

The present study shows a low prevalence of *E. coli* O157 in beef sold at butcher shops. Nevertheless, given the low infective dose of this pathogen and the deep-rooted tradition of consuming raw or undercooked beef, the current prevalence should not be considered lightly from a public health perspective.

## Background

In the 21^st^ century, foodborne diseases have become one of the important issues all over the world [1]. Due to poor infrastructure and low level of awareness, this problem is worse in developing countries [2]. Major pathogenic microorganisms that frequently have been associated with foods of animal origin include Enterohemorrhagic *Escherichia coli* O157. In humans this pathogen causes asymptomatic infection to severe diarrhea and/or hemolytic-uremic syndrome (HUS) [3]. Human infections with *E. coli* O157 have been mostly associated with the consumption of contaminated and improperly cooked minced beef and unpasteurized cow milk [4]. Butcher houses and restaurants are frequently incriminated as sources of *E. coli* O157 for human infections [5].

Ethiopia ranks second after Nigeria in the health burden of zoonotic diseases in Africa [6]. This country is located in a sub-region that experiences the second highest foodborne disease burden in the world, where *E. coli* O157 is one of the leading causes of foodborne disease disability adjusted life years [7]. In Ethiopia, years of life lost due to diarrheal diseases was 2.6 million in 2010, and diarrheal diseases are the second leading cause of premature death after lower respiratory infections [8]. Information about human infections with *E. coli* O157 is limited in this country, however, in a study conducted on 422 diarrheic children under 5 years in northern part of Ethiopia, *E. coli* O157:H7 was isolated from 59 (28.9%) of the children [9]. The habit of consuming raw and/or undercooked meat is one of the factors that exacerbate the transmission of foodborne pathogens including *E. coli* O157 in the country. Sufficient heating of meat kills these organisms [4]; however, in Ethiopia, most people prefer to eat raw or undercooked beef (locally called *kitfo*, *dulet*, and *kurt*).

Treatment of Enterohemorrhagic *E. coli* infections with antibiotics may worsen the illness, presumably by breaking up the bacteria with the release of more toxins and increased toxin production [10]. However, early administration using some antimicrobials is effective [11]. Unfortunately, inappropriate ways of antimicrobial uses have contributed to the increase in antimicrobial resistance [12].

In Ethiopia, only few small-scale studies estimating the prevalence and/or assessing the antimicrobial sensitivity profile of enterohemorrhagic *E. coli* O157 in feces, skin swabs and carcasses of sheep, goats, and cattle at abattoirs were conducted [13–16]. But studies at the level of consumption particularly in butcher shops and restaurants is lacking. Consequently, this study was designed to address the information gap pertaining to the prevalence and antibiotic susceptibility profiles of *E. coli* O157 in beef carcass and minced beef at butcher shops and restaurants respectively in central Ethiopia.

## Methods

### Study design and sample collection

From December 2013 to April 2014, carcass and carcass in-contact surface swabs of 55 randomly selected butcher shops, and minced beef samples of 40 randomly selected restaurants were collected in four cities (Addis Ababa, Bishoftu, Batu, and Holetta) in central Ethiopia.

These cities have municipal abattoirs which render slaughter services to their respective dwellers. Butcher shops and restaurants get their beef from cattle slaughtered in the abattoirs of their respective cities. Nevertheless, back yard or illegal slaughtering of animals is a common practice. The abattoirs are barely equipped with necessary facilities, and shortage of clean water is one of the chronic problems for most abattoirs in the country. In addition, the sanitary condition of most restaurants and butcher shops is poor; generally, such food catering firms are loosely monitored and regulated. In Ethiopia, fresh beef cuts are commonly purchased from butcher shops and are consumed at home or at the same butcher shop, either cooked or raw. In butcher’s shops, a beef carcass is kept in open-air at environmental temperature (in Addis Ababa 7 to 25 °C daily temperature).

The butcher shops and restaurants in each city were selected by a simple random sampling technique, using lists obtained from city administrations of the respective cities as sampling frames. When visiting each selected butcher shop and restaurant, the purpose of the study was explained to the manager, and a letter from the College of Veterinary Medicine and Agriculture Addis Ababa University demonstrated the approval and credibility of our work. From each butcher shop on average eight samples were collected on two separate days. A total of 440 swab samples consisting of 110 samples from beef carcass, knife, cutting board, and butcher’s hands at butcher shops, respectively, and 85 minced beef (locally also called *kitfo*) samples from restaurants were collected (Table 1). Beef carcasses were swabbed following the method described in ISO17604 (2003). A sterile cotton tipped swab (2X3cm) fitted with shaft, was first soaked in an approximately 10 ml of buffered peptone water (BPW; Oxoid, Hampshire, UK) and subsequently rubbed horizontally and vertically several times on the carcass surface. After the rubbing process, the shaft was broken by pressing it against the inner wall of the test tube leaving the cotton swab inside the test tube. The whole surface of the carcass was swabbed by one swab, and swabs from multiple carcasses from the same butcher shop were pooled together. Carcass in-contact surfaces including knife, butcher’s hands and cutting board swabs were obtained by swabbing them using a cotton swab pre-moistened in BPW. The junction of the handle and blade of all knives in each butcher shop was swabbed and pooled together. Similarly, both sides of the two hands of the butcher, and the whole surface of the wooden cutting board were swabbed. The carcass swab, the butcher’s hands swab, the cutting board swab and the knife swab were kept separately. The minced beef samples were taken from ready-to-eat undercooked (*n* = 45) and raw minced beef (*n* = 40) at the selected restaurants. All samples were transported in cold boxes to the laboratory. The samples were stored at 4 to 7 °C and analyzed within 6 to 12 h as described in ISO 16654:2001.

**Table 1:** *Escherichia coli* O157 isolated from carcass, hand, knife, and cutting board swabs and minced beef in central Ethiopia

	Number of positive samples/number of tested samples				
Sample types	Addis Ababa	Bishoftu	Batu	Holetta	Total
Carcass swab	1/25	2/30	1/25	1/30	5/110
Hand swab	0/25	0/30	0/25	0/30	0/110
Knife swab	0/25	0/30	0/25	0/30	0/110
Cutting board swab	1/25	1/30	0/25	2/30	4/110
Minced beef	0/20	0/25	0/20	0/20	0/85
Total	2/120	3/145	1/120	3/140	9/525

### Sample processing

Ninety ml of modified tryptone soy broth supplemented with novobiocin (mTSB + N; Oxoid) were added to 10 ml swab sample. Conversely, 25 g of each minced beef sample were collected in a Stomacher bag. After adding 225 ml mTSB + N, each sample was homogenized using a Stomacher 400 (Seward Medical, England) for two minutes and transferred into a sterile flask. After incubation at 41.5 ± 0.5 °C for 24 h, all enrichment broths were plated onto sorbitol MacConkey agar (Oxoid) supplemented with 0.05 mg/l cefixime and 2.5 mg/l potassium tellurite (Oxoid) (SMAC-CT) and incubated at 37 °C for 18 to 24 h. Multiple non-sorbitol fermenting typical *E. coli* colonies from a plate were streaked out on the SMAC-CT agar and incubated for 24 h at 37 °C for the confirmatory test. All colonies that did not ferment sorbitol on the SMAC-CT agar underwent a slide agglutination test using an *E. coli* O157 latex test kit (Oxoid) and were considered *E. coli* O157 positive when precipitation occurred within one minute.

### Antimicrobial susceptibility testing

The *E. coli* O157 isolates were tested for antimicrobial susceptibility to a panel of the following ten antimicrobial agents: amoxicillin (AMX 25 μg), kanamycin (KAN 30 μg), trimethoprim-sulfamethoxazole (SXT 25 μg), chloramphenicol (CHL 30 μg), ciprofloxacin (CPR 5 μg), streptomycin (STR 10 μg), nalidixic acid (NA 30 μg), cefoxitin (CFX 30 μg), tetracycline (TTC 30 μg), and nitrofurantoin (NTR 50 μg) using the disc diffusion method according to the guidelines for Clinical and Laboratory Standards Institute [17]. All test discs were obtained from Oxoid. The isolates were classified as sensitive, intermediate, or resistant using the breakpoints of the CLSI. The standard reference strain of *E. coli* ATCC 25922, sensitive to all tested antimicrobial agents, was used as the control strain.

### Statistical analysis

The data were analyzed using STATA Version 11.0 (STATA corp. College Station, TX, USA). Descriptive statistics (estimation of proportions) were used to summarize the prevalence of *E. coli* O157 and antimicrobial sensitivity patterns of the isolates.

## Results

### *E. coli* O157 isolated from butcher shops and restaurants

Out of the 525 collected samples, 60 of them had *E. coli* O157 suspect colonies on the CT- SMAC plates (40 samples from the butcher shops and 20 samples from the restaurants). Only nine (1.7%, 95% CI: 0.8–3.3%) of the 525 examined samples were confirmed to be positive for *E. coli* O157 (Table 1). Two samples (one carcass swab and one cutting board swab) obtained from the same butcher shop during the same visit were positive for *E. coli* O157, but in the other cases, the positive carcass swabs and the positive cutting boards swabs were from different butcher shops. *E. coli* O157 was not detected in any of the minced beef samples.

### Antimicrobial susceptibility profiles of *E. coli* O157 isolates

All isolates were susceptible (100%) to five of the ten antimicrobial agents (trimethoprim-sulfamethoxazole, tetracycline, cefoxitin, kanamycin, and nalidixic acid; Table 2). Conversely, 5, 3, and 2 of the isolates were resistant to amoxicillin, streptomycin, and chloramphenicol, respectively. Only two of the isolates were susceptible to all examined antimicrobial agents. One isolate from the carcass was resistant to amoxicillin and chloramphenicol, and one isolate from the cutting board was resistant to amoxicillin, chloramphenicol, and streptomycin.

**Table 2:** Antimicrobial susceptibility profiles of nine *Escherichia coli* O157 isolates of carcass and cutting board samples collected from butcher shops in four cities in central Ethiopia

Antimicrobial agents	Holetta			Bishoftu			Addis Ababa	Batu	
	Ca^a^	Ca	CB^a^	Ca	Ca	CB	Ca	CB	Ca
Amoxicillin	I^b^	I	I	R^b^	I	R	R	R	R
Cefoxitin	S^b^	S	S	S	S	S	S	S	S
Chloramphenicol	S	S	S	R	I	R	S	S	S
Ciprofloxacin	S	S	S	S	S	I	S	S	I
Kanamycin	S	S	S	S	S	S	S	S	S
Nalidixic acid	S	S	S	S	S	S	S	S	S
Nitrofurantoin	S	S	S	S	S	S	I	S	I
Streptomycin	S	S	R	I	R	R	S	I	I
Tetracycline	S	S	S	S	S	S	S	S	S
Trimethoprim-Sulfamethoxazole	S	S	S	S	S	S	S	S	S

^a^Sample types: *Ca* carcass, *CB* cutting board; ^b^Antimicrobial susceptibility patterns, *R* resistant, *I* intermediate resistant, *S* susceptible

## Discussion

Food borne infections are major health concerns in developing countries including Ethiopia. Information on incidence of these infections and their susceptibility to antimicrobials helps policy makers to develop appropriate strategies in terms of prevention, treatment and control. In this study, 4.5% (5/110) carcass swabs were positive for *E. coli* O157. Tendencies for higher prevalences were observed in previous studies. In one of the studies, eight of 86 beef samples (i.e. 9.3%) collected from butcher shops in central Ethiopia were positive [14]. Hiko et al. [14] examined meat samples while we tested swabs of carcass surfaces and carcass in-contact surfaces. Likewise, Mersha, Asrat [18] found at an export abattoir in central Ethiopia that 8.1% (14/172) of sheep and goat carcass surface swabs taken before washing and 8.7% (15/172) of carcass surface swabs after washing were contaminated by *E. coli* O157:H7. Also, the prevalence of *E. coli* O157 in the carcass surface swabs of our study (4.5%) was similar to reports from eastern Ethiopia (2.65%, 3/113 beef carcass surface swabs) at Haramaya University slaughter house [16] and from Turkey (2%, 2/100 beef carcass surface swabs) at two commercial abattoirs in Samsun Province [19], and from the UK (2.9%, 29/1877 samples of lamb products) at butcher shops in South Yorkshire. However, the prevalence in beef products at the same butcher shops tended to be lower (1.1%, 36/3216 samples of beef products) [3]. Nevertheless differences in prevalences could have been due to the limited sample size in our and several of the other studies.

In this study, *E. coli* O157 was isolated from 3.6% (4/110) of the surface swabs of wooden cutting boards; however, none of the swabs from butcher hands and knives were positive. In a similar kind of study conducted in Pakistan, *E. coli* O157:H7 was not detected in surface swabs (knives, wooden boards, weighing scales, and meat mincers) taken from 30 individual retail meat outlet markets [20]. In our study, in one case both the beef carcass surface swab and the cutting board swab collected during the same visit to the same butcher shop were positive, indicating the possible contamination of the wooden board by the carcass or vice versa; however, in other three cases, *E. coli* O157 was not detected in the carcass samples from butcher shops where the cutting boards were positive. The isolation of *E. coli* O157 from the carcass in-contact material although the carcass itself was negative, may suggest the presence of other potential sources of contamination in butcher shops like cleaning water or inadequate cleaning and disinfection of the cutting boards leading to possible biofilm formation by the organisms on the wooden board. Indeed, *E. coli* O157 has been isolated from water samples in Ethiopia [13, 18] and biofilm formation of *E. coli* O157 in various food contact surfaces and tolerance to sanitizing reagents has been reported [21, 22]. *E. coli* O157 contaminated cutting boards can be an important source of cross contamination and may pose a significant public health risk.

*E. coli* O157 was not detected in the 85 minced beef samples from the 40 restaurants, which is in agreement with reports from Turkey [23] and Seattle, USA [24]. By contrast, 11.25% of cooked beef doner kebabs in Turkey [25] and 3.8% of minced beef in Argentina [26] were contaminated by *E. coli* O157:H7. In our study, the inability of isolating *E. coli* O157 from the ground beef samples where the contamination of beef carcass at butcher shops and abattoirs is not uncommon could be attributed to the way *kitfo* is prepared. Minced beef is seasoned with butter and other spices to prepare *kitfo*. Antimicrobial effects of some Ethiopian spices against *E. coli* and other organisms have been demonstrated [27]. The spice blends may stress the organisms, which may make the isolation of *E. coli* O157 from contaminated samples difficult. However, the stressed organisms retain their pathogenicity and thus pose risk to humans, given the fact that *E. coli* O157 has a low infective dose [11]. The small number of samples considered in the current study calls for a further large scale study to estimate the real prevalence of *E. coli* O157 in *kitfo,* and to investigate whether the *kitfo* preparation process has a possible antibacterial effect. Before such a study can be done an evaluation of the efficiency of the applied detection method to isolate possible sub-lethal injured *E. coli* O157, caused by spices in such beef product, is recommended.

In the present study some of the isolates were resistant to amoxicillin (*n* = 5), streptomycin (*n* = 3), and chloramphenicol (*n* = 2). While previous studies from Ethiopia and other countries reported resistance to amoxicillin and/or streptomycin, the resistance observed against chloramphenicol in the current study is in contrast to the previous reports of 100% susceptibility to this antibiotic [13, 14, 16, 28]. On the other hand, resistance to chloramphenicol and other drugs have been reported elsewhere [29, 30]. In the present study, one isolate was resistant to two antimicrobials (amoxicillin and chloramphenicol) and another isolate to three antimicrobials (amoxicillin, chloramphenicol, and streptomycin), which is in agreement with previous studies [13, 14, 16, 29, 31]. In current study, a low level of antimicrobial resistance was observed in comparison to previous studies; this might be related to the fewer number of antimicrobial panels we used. However, the current finding has a significant public health implication.

Among the antimicrobials we examined, trimethoprim-sulfamethoxazole is one of the β-lactams antibiotics that their prescription for *E. coli* O157:H7 infections is debatable, as these drugs may surge the risk of HUS in children [32–34]. On the other hand, another study shows that in the early stage of infections these antibiotics do not entail risk [35]. Some of the drugs we tested such as amoxicillin, cifoxitin, and trimethoprim-sulfamethoxasole can increase the risk of HUS; and thus they are not recommended for the treatment of infections caused by *E.coli* O157:H7. Fluoroquinolones family drugs such as ciprofloxacin and nalidixic acid are commonly prescribed for adults but not for children. However, two of our isolates showed intermediate resistant to ciprofloxacin which is alarming.

## Conclusions

In conclusion, the present study demonstrates low prevalence of *E. coli* O157 (1.7%) in butcher shops in central Ethiopia. Nevertheless, given the low infective dose of *E. coli* O157 and the deep-rooted tradition of consuming raw or undercooked beef in the society, the current prevalence should be considered important from a public health perspective and surveillance to monitor *E. coli* O157 and other pathogens in butcher shops should be organized. *E. coli* O157 was detected on cutting boards in four butcher shops, indicating that such equipment can function as a source for contamination of beef. In order to prevent such contamination butchers have to improve their hygiene practice. *E. coli* O157 was not detected in the *kitfo* samples from restaurants. Presence of spices in *kitfo* may lead to sub-lethal *E. coli* O157 cells leading to false-negative results. The isolates were susceptible to most of the drugs used in the in vitro test; however, the resistance observed against chloramphenicol in the current study is in contrast to the previous three studies in Ethiopia.
